# Data transformation of unstructured electroencephalography reports by natural language processing: improving data usability for large-scale epilepsy studies

**DOI:** 10.3389/fneur.2025.1521001

**Published:** 2025-02-28

**Authors:** Yoon Gi Chung, Jaeso Cho, Young Ho Kim, Hyun Woo Kim, Hunmin Kim, Yong Seo Koo, Seo-Young Lee, Young-Min Shon

**Affiliations:** ^1^Department of Pediatrics, Seoul National University Bundang Hospital, Seoul National University College of Medicine, Seongnam-si, Gyeonggi-do, Republic of Korea; ^2^Department of Pediatrics, Seoul National University College of Medicine, Seoul, Republic of Korea; ^3^Department of Neurology, Asan Medical Center, University of Ulsan College of Medicine, Seoul, Republic of Korea; ^4^Department of Neurology, Kangwon National University School of Medicine, Chuncheon-si, Republic of Korea; ^5^Interdisciplinary Graduate Program in Medical Bigdata Convergence, Kangwon National University, Chuncheon-si, Republic of Korea; ^6^Department of Neurology, Samsung Medical Center, Sungkyunkwan University School of Medicine, Seoul, Republic of Korea

**Keywords:** natural language processing, electroencephalography, epilepsy, deep learning, keyword extraction

## Abstract

**Introduction:**

Electroencephalography (EEG) is a popular technique that provides neurologists with electrographic insights and clinical interpretations. However, these insights are predominantly presented in unstructured textual formats, which complicates data extraction and analysis. In this study, we introduce a hierarchical algorithm aimed at transforming unstructured EEG reports from pediatric patients diagnosed with epilepsy into structured data using natural language processing (NLP) techniques.

**Methods:**

The proposed algorithm consists of two distinct phases: a deep learning-based text classification followed by a series of rule-based keyword extraction procedures. First, we categorized the EEG reports into two primary groups: normal and abnormal. Thereafter, we systematically identified the key indicators of cerebral dysfunction or seizures, distinguishing between focal and generalized seizures, as well as identifying the epileptiform discharges and their specific anatomical locations. For this study, we retrospectively analyzed a dataset comprising 17,172 EEG reports from 3,423 pediatric patients. Among them, we selected 6,173 normal and 6,173 abnormal reports confirmed by neurologists for algorithm development.

**Results:**

The developed algorithm successfully classified EEG reports into 1,000 normal and 1,000 abnormal reports, and effectively identified the presence of cerebral dysfunction or seizures within these reports. Furthermore, our findings revealed that the algorithm translated abnormal reports into structured tabular data with an accuracy surpassing 98.5% when determining the type of seizures (focal or generalized). Additionally, the accuracy for detecting epileptiform discharges and their respective locations exceeded 88.5%. These outcomes were validated through both internal and external assessments involving 800 reports from two different medical institutions.

**Discussion:**

Our primary focus was to convert EEG reports into structured datasets, diverging from the traditional methods of formulating clinical notes or discharge summaries. We developed a hierarchical and streamlined approach leveraging keyword selections guided by neurologists, which contributed to the exceptional performance of our algorithm. Overall, this methodology enhances data accessibility as well as improves the potential for further research and clinical applications in the field of pediatric epilepsy management.

## 1 Introduction

Electroencephalography (EEG) is a noninvasive diagnostic modality that is specifically designed to record neuronal activity within the brain. This technique has demonstrated considerable efficacy in identifying clinically significant cortical electrophysiological markers in individuals suffering from neurological disorders. As such, EEG assessments serve as preliminary diagnostic measures for neurological conditions, especially epilepsy (1–3).

Typically, EEG reports are composed of unstructured data formatted as free-text, which varies in stylistic presentation depending on the individual neurologist. This variability necessitates that neurologists must manually scrutinize each report to aggregate the data into a cohesive database—a process that is both time-consuming and labor-intensive. Consequently, the potential for large-scale data analysis and related clinical applications utilizing EEG reports has been significantly curtailed. Despite these reports containing valuable clinical insights vital for the interpretation of patient EEG recordings, no systematic efforts have been achieved to convert this information into structured, tabulated datasets. In response to these challenges, recent advancements in natural language processing (NLP) techniques have emerged as promising solutions for managing unstructured data in electronic medical records (EMR) and identifying information from text-heavy EEG reports of epilepsy patients (4). Various methodologies involving rule-based systems, machine learning algorithms, and deep learning approaches have been implemented for a spectrum of tasks such as information extraction, text classification, and summarization (4–6). These innovations offer the potential to revolutionize the handling of EEG reports, thereby enhancing their utility in clinical and research settings.

Despite the significant advancements in information extraction and text classification, the majority of existing studies have primarily focused on clinical notes and discharge summaries instead of EEG reports. Researchers have utilized both rule-based and deep learning methodologies to extract epilepsy- and seizure-related variables from these free-text documents. The key variables include epilepsy phenotypes (7, 8), seizure onsets (9), seizure frequency (8, 10, 11), seizure types (8, 10, 12), and EEG patterns (13). Additionally, certain studies have focused on classifying patients based on their seizure-free status (11, 12). However, the extensive array of target variables for keyword extraction from clinical notes and summaries presents challenges in data selection strategies when applying NLP techniques. In contrast, EEG reports typically offer more concise and focused information, particularly regarding the electrographic findings of patients. This structured format allows for effective analysis in a time-sequenced manner when processed sequentially.

Therefore, in the present study, we propose a hierarchical algorithm designed to transform unstructured EEG reports from pediatric patients diagnosed with epilepsy into structured data that is clinically relevant, leveraging advanced NLP techniques. This algorithm was designed to achieve the following objectives: (1) convert large volumes of free-text EEG reports into tabular data using deep learning and simplified rule-based methods with high accuracy and (2) ensure easy adaptability to various EEG report formats through external validation.

## 2 Methods

### 2.1 Dataset

We retrospectively compiled 17,172 reports from 3,423 pediatric patients (mean age: 10.8 ± 6.0 years) diagnosed with epilepsy. These reports were sourced from the clinical data warehouse of Seoul National University Bundang Hospital (SNUBH), situated in Seongnam, Republic of Korea. Two neurologists, identified as H.K. and J.C., meticulously reviewed all the EEG reports to categorize them as either normal or abnormal. A report was deemed normal if it revealed no abnormal findings, whereas an abnormal report was characterized by the presence of at least one abnormal finding. Based on the annotations of the neurologists, we identified 6,173 reports as normal and 10,822 as abnormal. To facilitate a balanced dataset, we randomly selected 6,173 abnormal reports to match a 1:1 ratio of normal to abnormal cases. For the purposes of developing our classification algorithm, we further narrowed our selection to 5,173 reports from both the normal and abnormal categories. The remaining 1,000 reports from each category were reserved for the evaluation of our classification models and internal validation.

**Figure 1:**
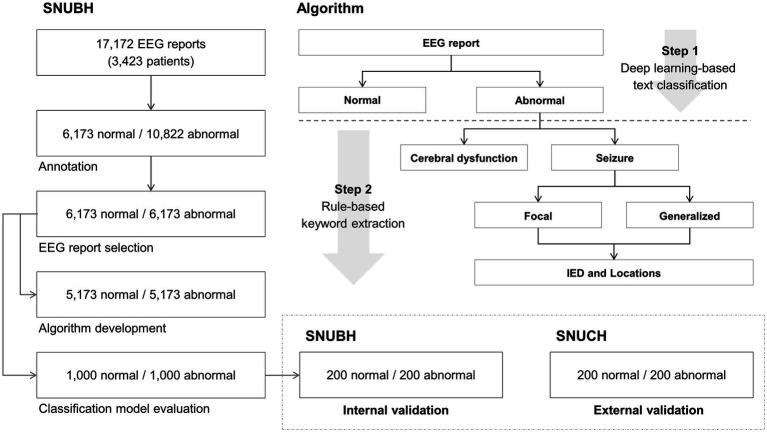
Overall process of this study. Normal and abnormal electroencephalography (EEG) reports (5,173 each) from the Seoul National University Bundang Hospital (SNUBH), located in Seongnam, Republic of Korea, were used for algorithm development. Normal and abnormal reports (1,000 each) of SNUBH were used for classification model evaluation. Among the 1,000 normal and 1,000 abnormal reports, randomly selected 200 normal and 200 abnormal reports were used for internal validation. Normal and abnormal reports (200 each) from an independent tertiary hospital in Korea (Seoul National University Children’s Hospital (SNUCH), Seoul, Republic of Korea) were used for external validation. IED denotes interictal epileptiform discharge.

Additionally, we conducted a retrospective collection of 400 EEG reports from 229 pediatric patients (age: 9.3 ± 7.8 years) with epilepsy, sourced from the clinical data warehouse of Seoul National University Children’s Hospital (SNUCH), which is an independent tertiary facility located in Seoul, Republic of Korea. The same neurologists, H.K. and J.C., reviewed these 400 reports and confirmed that they were 200 normal and 200 abnormal reports. All EEG reports from SNUCH were employed for the external validation. The overall study process is illustrated in Figure 1.

This research was granted approval by the Institutional Review Board at Seoul National University Bundang Hospital (Approval No. B-2312-873-107). Due to the retrospective nature of the study, the requirement for informed consent was waived. The research adhered to the ethical principles outlined in the Declaration of Helsinki.

### 2.2 Algorithm development

We executed a two-step process to transform the free-text EEG reports into structured tabular data. The first step involved classifying the EEG reports into two categories: normal and abnormal, using a deep learning-based model. The primary aim of this classification was to identify the abnormal EEG reports, which encapsulate critical findings from neurologists concerning various abnormalities. The second step focused on extracting specific keywords from the identified abnormal EEG reports using rule-based methodologies. The main goal of this keyword extraction was to pinpoint significant abnormal findings that could provide insights into the condition of patients diagnosed with epilepsy. This second step comprised three sequential procedures for keyword extraction. Below, we provide a comprehensive overview of our hierarchical algorithm:

(1) Step 1: Classification of normal and abnormal reports.

EEG reports were categorized as either normal or abnormal through the application of a deep learning-based classification model. Reports classified as normal did not proceed to further analysis. Conversely, those identified as abnormal prompted the execution of the second phase, as detailed below. Note that abnormal reports may include keywords that suggest both *normal* and *abnormal* conditions (e.g., “*This is a normal waking and moderately abnormal stage I-II*…”). In cases where a report contained solely *abnormal* keywords, it was categorized as abnormal irrespective of the model’s output.

(2) Step 2: Keyword extraction.

The extraction of specific keywords from the abnormal EEG reports was performed using rule-based methods that relied on regular expressions and the spaCy library in Python. This method facilitated the identification of relevant keywords that denote significant abnormal findings. All abnormal reports were structured into two distinct sections: impression and clinical correlation. Initially, each abnormal report was divided into these two sections, and one section was selected based on the targeted keywords. The extraction of keywords was conducted through a series of three hierarchical procedures, detailed as follows:

- Extraction of keywords related to *dysfunction* or *seizure* from the clinical correlation section: In instances where an abnormal report indicated *dysfunction*, it was inferred that the corresponding background EEG activity was abnormal (e.g., *cerebral dysfunction* or *occipital lobe dysfunction*). Conversely, if the report identified *seizure* activity, it was determined that the corresponding EEG exhibited characteristics indicative of a seizure. Both keywords, *dysfunction* and *seizure*, were systematically extracted.
- Extraction of *focal* (or *partial*) or *generalized* seizure information from the clinical correlation section: This procedure was specifically applied to abnormal reports identified in the previous procedure (A) that indicated *seizure* activity. If the report contained references to *focal* (or *partial*) seizures, the corresponding seizure type was classified as focal; if it referenced *generalized* seizures, the classification was adjusted accordingly to generalized. Both keywords were extracted to ensure comprehensive categorization.
- Extraction of keywords associated with interictal epileptiform discharges (IEDs) and their respective locations from the impression section: This procedure was applied to the abnormal reports identified in the earlier step (A) that contained *seizure* activity. We defined keywords relating to IEDs as any phrases incorporating the terms *spike*, *discharge*, *wave*, *sharp*, or all possible combinations of these four terms (such as *spike discharge* or *sharp wave discharge*). The keywords related to the locations of IEDs were defined as phrases with one or all possible combinations of the names of the 19 channels according to the international 10–20 system (e.g., *Fp1* or *F3F7*).

### 2.3 Deep learning model

In the first phase of our algorithm development, we established deep learning-based binary classification models aimed at categorizing EEG reports as either normal or abnormal. For this purpose, we employed two publicly available language models from the Hugging Face repository: Bidirectional Encoder Representations from Transformers (BERT) and Clinical BERT. BERT is a transformer-based deep learning model pretrained on extensive datasets such as BooksCorpus and Wikipedia. In contrast, Clinical BERT is a specialized variant of BERT, pretrained on clinical text corpora, which includes clinical notes extracted from the MIMIC-III database (14–17).

We selected the BERT-base model from Hugging Face, characterized by 12 transformer layers, hidden size of 768, 12 self-attention heads, and a total parameter count of 110 million, which aligns with the specifications of Clinical BERT. Both the BERT and Clinical BERT models were fine-tuned using an equal dataset composed of 5,173 normal reports and 5,173 abnormal reports to optimize their performance in classification tasks. Each report was tokenized with a maximum length limit of 128 tokens before feeding to the input layer of the model. No additional preprocessing was applied to the reports. AutoTokenizer from Hugging Face tokenized all the reports yielding input IDs, token type IDs, and attention mask value sets for each report. We used zero padding to the maximum length of tokens and truncation to provide data sets for the input layer. Supplementary Table 1 shows an example of a tokenized EEG report.

To augment the capability of the model in sentence recognition, we concatenated each model with long short-term memory (LSTM) networks with both BERT and Clinical BERT, resulting in two enhanced architectures: BERT with LSTM and Clinical BERT with LSTM. In these configurations, the output generated from the final hidden layer of each model was subsequently directed into the input layer of the LSTM, thereby creating a cohesive model that leverages the strengths of both deep learning frameworks for improved classification outcomes. Supplementary Figure 1 shows our model architectures.

We used 64 LSTM units, a dropout rate of 0.1, and a sigmoid activation function, which yielded a probability score between 0 and 1. Specifically, an input report was classified as normal if the output was less than 0.5 and as abnormal if it was equal to or greater than 0.5. We used adaptive moment estimation as an optimizer with a learning rate of 1 × 10^−5^, a binary cross entropy loss function, a batch size of 32, and 5 epochs for model training. All algorithmic processes were executed using Python 3.8 and Tensorflow 2.10, facilitated by an NVIDIA 3080Ti graphics processing unit with 12GB of memory, in conjunction with the Compute Unified Device Architecture (CUDA) version 11.4 programming interface.

### 2.4 Performance evaluation

The performance of our algorithm was rigorously assessed through three key methodologies: (1) model evaluation, (2) internal validation, and (3) external validation. During the model evaluation phase, we examined the performance of our two deep learning models in classifying reports as normal or abnormal, using a dataset comprising 1,000 normal and 1,000 abnormal EEG reports sourced from SNUBH. The evaluation metrics included sensitivity, specificity, accuracy, and the area under the receiver operating characteristic curve (AUC). Internal and external validations were subsequently conducted to ascertain the applicability of the algorithm within a clinical setting, where EEG reports were systematically converted into structured tabular data from a clinical perspective. For the internal validation, we randomly selected 200 normal and 200 abnormal reports from the previously mentioned model evaluation dataset. In contrast, the external validation utilized a separate set of 200 normal and 200 abnormal reports from SNUCH. Two neurologists (H.K. and J.K.) compared 400 reports from SNUBH and 400 reports of SNUCH with their corresponding algorithm outputs in terms of all hierarchical procedures for internal and external validations, respectively. We adopted the Clinical BERT with LSTM model for normal and abnormal classifications in the internal and external validations. For the performance of the Clinical BERT with LSTM model, we additionally performed 6-fold cross-validation using the EEG reports from SNUBH. 1,000 normal and 1,000 abnormal reports were used for evaluation and the remaining ones were used for model training in each round.

## 3 Results

### 3.1 Model evaluation

In our evaluation of the classification models for distinguishing between normal and abnormal EEG reports from SNUBH, both the BERT with LSTM and Clinical BERT with LSTM models demonstrated impressive performance metrics: sensitivity of 100%, specificity of 99.90%, accuracy of 99.95%, and an AUC of 100%. Notably, each model produced one false-positive result. The outputs of the models, when averaged over 1,000 normal reports, were recorded as 0.173 ± 2.410% for the BERT with LSTM and 0.175 ± 3.137% for the Clinical BERT with LSTM. In contrast, the model outputs averaged over 1,000 abnormal reports were significantly higher, with the BERT with LSTM yielding 99.854 ± 0.175% and the Clinical BERT with LSTM achieving 99.870 ± 0.656%. Additionally, our Supplementary Table 2 includes detailed outputs from the Clinical BERT with LSTM model alongside the corresponding keyword extraction results for both 1,000 normal and 1,000 abnormal EEG reports. Supplementary Table 3 shows confusion matrices over 6-fold cross-validation of the Clinical BERT with LSTM model with the average sensitivity, specificity, accuracy, and AUC of 99.88, 99.98, 99.93, and 100%, respectively.

### 3.2 Internal validation

During the internal validation using the EEG reports from SNUBH, we attained a perfect accuracy of 100% in Step 1 for the classification of normal and abnormal EEG reports. Furthermore, in Step 2, the accuracy rates for the keyword extraction processes were also commendable, with 100% accuracy for classification A (*dysfunction* or *seizure*), 98.50% for classification B (*focal* or *generalized*), and 97.50% for classification C (IEDs and locations). Among the 200 abnormal EEG reports, we failed to extract the *generalized* information because the relevant keyword did not exist in the clinical correlation part in two reports, and in one report, the keyword was misspelled. Furthermore, we encountered challenges in extracting the locations of IEDs, because these characteristics were not specified as channel names in four different reports. Conversely, the model mistakenly identified a channel name that referred to locations of abnormal background activities in one of the reports.

### 3.3 External validation

During the external validation process utilizing EEG reports from the SNUCH, we achieved a perfect accuracy rate of 100% in Step 1 for classifying EEG reports as normal or abnormal. In Step 2, we recorded accuracy rates of 100, 100, and 88.50% for our keyword extraction procedures labeled A, B, and C, respectively. Among the 200 abnormal EEG reports analyzed from SNUCH, we were unable to extract the locations of IEDs in six reports due to the absence of channel name representation. Additionally, we erroneously extracted channel names indicative of abnormal background activities in 16 reports. In one instance, we mistakenly classified *delta waves*, described in a phrase concerning background activity, as an IED.

The detailed results from both internal and external validations are presented in Table 1. Furthermore, Tables 2, 3 show representative abnormal EEG reports from the SNUBH and SNUCH, respectively, highlighting both the successful and erroneous conversions into structured data during our validation process.

**Table 1:** Detailed results of the internal and external validations using the electroencephalography (EEG) reports from Seoul National University Bundang Hospital (SNUBH) and an independent tertiary hospital (SNUCH), respectively.

		Step 1	Step 2		
			A	B	C
SNUBH	I. Normal	200	-	-	-
	II. Abnormal	200	200	197	195
	Accuracy (%)	100	100	98.50	97.50
SNUCH	I. Normal	200	-	-	-
	II. Abnormal	200	200	200	177
	Accuracy (%)	100	100	100	88.50

A, B, and C in Step 2 denote the keyword extraction procedures for *dysfunction* or *seizure*, *focal (partial)* or *generalized* seizure, and the existence of interictal epileptiform discharges and their locations, respectively. I and II in the second column represent the number of EEG reports that are correctly converted to structured data for the Step 1 and Step 2.

**Table 2:** Representative electroencephalography (EEG) reports from Seoul National University Bundang Hospital (top three rows) and an independent tertiary hospital (bottom three rows) that are correctly converted to structured data evaluated by two neurologists in the internal and external validations, respectively.

EEG report	Step 1	Step 2				Model output
		A	B	C		
(Impression) This is a normal waking and mildly abnormal stage N1-2 sleep record due to a few low-voltage spike discharges from O2O1, during sleep. Clinical correlation: this recording is suggestive of focal seizure (subtle axial myoclonus without EEG changes was noted).	Abnormal	Seizure	Focal	Spike discharges	O2O1	0.9991
(Impression) This is a moderately abnormal waking and stage I-II sleep record due to: (1) Poorly regulated posterior rhythm for age. (2) High amplitude irregular 1.5–2 Hz delta slowing on both posterior head region. (3) Frequent spike discharge from C3P3T3 or F8T4 activated by sleep. Clinical correlation: This record is indicative of diffuse cerebral dysfunction and consistent with partial seizure.	Abnormal	Dysfunction, seizure	Partial	Spike discharge	C3P3T3 or F8T4	0.9991
(Impression) This is a mildly abnormal sedated sleep record due to intermittent medium to high amplitude 2.5–3 Hz delta activities on the anterior head region. Clinical correlation: This recording is indicative of anterior cerebral dysfunction.	Abnormal	Dysfunction				0.9990
This is a mildly abnormal Stage II sleep record due to a few low voltage spike discharges from F3C3P3 or P4T4. Clinical correlation: this recording is consistent with focal seizure.	Abnormal	Seizure	Focal	Spike discharges	F3C3P3 or P4T4	0.9992
This is a moderately abnormal Stage II sleep record due to: (1) Frequent generalized polyspike wave discharges or paroxysmal fast activities. (2) Frequent spike discharges form C3T3 or C4T4. Clinical correlation: this recording is consistent with focal and generalized seizure.	Abnormal	Seizure	Focal and generalized	Polyspike wave discharges, spike discharges	C3T3 or C4T4	0.9991
This is a moderately abnormal drowsy and sleep record due to: (1) Intermittent delta activities on the anterior head region. (2) Frequent low to medium voltage spike or spike wave discharges from Fp1F3F7 and Fp2F4F8. Clinical correlation: this recording is suggestive of diffuse cerebral dysfunction and consistent with focal seizure.	Abnormal	Dysfunction, seizure	Focal	Spike or spike wave discharges	Fp1F3F7 and Fp2F4F8	0.9991

A, B, and C in Step 2 denote the keyword extraction procedures for *dysfunction* or *seizure*, *focal (partial)* or *generalized* seizure, and the existence of interictal epileptiform discharges and their locations, respectively. The model output represents the probability from 0 to 1 that its corresponding report is determined as a normal one if the model output <0.5 and as an abnormal one if the model output ≥0.5.

**Table 3:** Representative electroencephalography (EEG) reports from Seoul National University Bundang Hospital (top four rows) and Seoul National University Children’s Hospital (bottom three rows) that are incorrectly converted to structured data evaluated by two neurologists in the internal and external validations, respectively.

EEG report	Step 1	Step 2				Model output
		A	B	C		
(Impression) This is a moderately abnormal waking and stage I-II sleep record due to: (1) Frequent episodes of generalized rhythmic 3 Hz spike wave discharges with videographic evidence of dialeptic seizure. (2) Occasional generalized spike wave discharges Clinical correlation: this recording is diagnostic of electroclinical absence seizure.	Abnormal	Seizure		Spike wave discharges (2)		0.9990
(Impression) This is a mildly abnormal waking and normal stage I-II sleep record due to two episodes of brief, rhythmic, bifrontal, 3 Hz, rhythmic delta activities (which cannot be discriminated from typical 3 Hz spike wave discharges - video is not available.) Clinical correlation: this recording is suggestive of generalized seizure.	Abnormal	Seizure		Spike wave discharges		0.9990
(Impression) This is a moderately abnormal sedated sleep record due to: (1) Diffuse high amplitude irregular pleomorphic 1.5–2.0 Hz delta activities. (2) Frequent spike discharges from the left or right centro-temporal area. Clinical correlation; this record is indicative of diffuse cerebral dysfunction and consistent with partial seizure (modified hypoarrythmia).	Abnormal	Dysfunction, seizure	Partial	Spike discharges		0.9991
(Impression) This is a mildly abnormal waking and normal stage N1-2 sleep record due to brief, intermittent, high amplitude, 2–3 Hz rhythmic delta activities from both posterior head region or P4O2, during and after hyperventilation. Clinical correlation: this recording is suggestive of both posterior cerebral dysfunction worse on the right hemisphere.	Abnormal	Dysfunction			P4O2	0.9990
This is a moderately abnormal drowsy and Stage I-II sleep record due to: (1) Intermittent delta activities on T6O2 during drowsiness. (2) A few or occasional spike discharges from T4T6 Clinical correlation: this recording is suggestive of left temporo-occipital cerebral dysfunction and consistent with focal seizure	Abnormal	Dysfunction, seizure	Focal	Spike discharges	T6O2, T4T6	0.9991
This is a mildly abnormal sleep record due to a few atypical spike discharges from the right or left frontocentral areas. Clinical correlation: this recording is suggestive of focal seizure	Abnormal	Seizure	Focal	Spike discharges		0.9991
This is a moderately abnormal record due to: (1) Medium to high delta waves in right hemisphere. (2) Slowing in both hemisphere. Clinical correlation: this recording is indicative of diffuse cerebral dysfunction.	Abnormal	Dysfunction		Waves		0.9986

A, B, and C in the Step 2 denote the keyword extraction procedures for *dysfunction* or *seizure*, *focal (partial)* or *generalized* seizure, and the existence of interictal epileptiform discharges and their locations, respectively. The model output represents the probability from 0 to 1 that its corresponding report is determined as a normal one if the model output <0.5 and as an abnormal one if the model output ≥0.5.

## 4 Discussion

The present findings confirm that the NLP-based hierarchical algorithm we developed effectively classified free-text EEG reports from pediatric patients diagnosed with epilepsy as either normal or abnormal. The algorithm demonstrated its capability to identify the presence of cerebral dysfunction or seizures within the abnormal reports. We demonstrated that our algorithm converted abnormal reports to tabular data with an accuracy higher than 98.5% for the determination of focal or generalized seizures and higher than 88.5% for the identification of IEDs and their locations. Neurologists identified a set of clinical keywords essential for the diagnosis of epilepsy prior to the analysis. Following this, we systematically extracted keywords from abnormal reports through a series of methodical procedures. Accordingly, we successfully developed structured datasets that accurately correspond to the EEG reports obtained from two distinct medical institutions.

### 4.1 Normal and abnormal classification

In Step 1 of our algorithm, we implemented a deep learning-based classification model designed specifically to identify abnormal EEG reports for subsequent keyword extraction tasks. Previous studies have demonstrated that BERT-based classification models perform exceptionally well in text classification across various medical domains (11, 18–20). Therefore, we were optimistic that we could apply our detailed rules for keyword extraction exclusively to the abnormal reports once we amassed a sufficient quantity of these datasets. Additionally, in the deep learning-based classification, we expected to avoid two situations: skipping required rules due to misspelled *abnormal*, or executing unnecessary rules due to misspelled *normal* in the reports. If we had utilized only the rule-based classification approach in Step 1, we would have faced a considerable risk of misclassifying reports, as misspellings in both *abnormal* and *normal* reports could easily lead to false recognition, and thus, erroneous classification of normal and abnormal reports.

As most normal EEG reports contain general words describing waking and sleep states, our BERT and Clinical BERT models had no additional domain-specific fine-tuning, unlike previous studies (11, 20, 21). We fine-tuned our models for the binary classification of normal and abnormal reports. Both BERT and Clinical BERT models exhibited high performance for binary classification, probably because the properties of normal and abnormal reports were highly different from each other in that the abnormal reports contained a significantly higher frequency of epilepsy-related terminology when compared to their normal counterparts. Another reason of the similar performance of the two models to each other may have been arisen from the data sources of the Clinical BERT. The MIMIC-III database contained a large number of clinical text data across various diseases. However, its knowledge in the field of epilepsy could be possibly weak because it handled less amount of data for neurological diseases and EEG examinations (22). During our evaluation, we encountered a single false positive for both models, an occurrence linked to the unique sentence structure of the report in question. This structure deviated substantially from that of typical normal reports, as it included enumerated numerical values regarding background activity rather than descriptive sentences detailing the state of normal EEG recordings.

Notably, we observed that the output scores from the Clinical BERT model were marginally higher than those from the BERT model for abnormal reports. Although this difference did not reach statistical significance, we hypothesized that the Clinical BERT model might inherently be predisposed to assign higher probabilities to abnormal reports than the BERT model. This observation prompts the necessity for further research to validate our hypothesis. Based on these findings, we opted to utilize the Clinical BERT model for Step 1 in both our internal and external validation processes.

### 4.2 Keyword extraction

In Step 2 of our algorithm, we systematically implemented a series of rules to extract specific keywords from abnormal EEG reports. We argue that a rule-based approach to keyword extraction is justified, as a finite set of key terms can effectively capture the defining features of abnormal EEG reports. This assertion is supported by prior research that examined the limitations of BERT in this context (23). Moreover, we aimed to enhance the transparency of the keyword extraction process in Step 2 by utilizing explicit rules, which aligns with the previous studies that underscored concerns regarding reproducibility in machine learning models (4, 24).

Rule-based techniques enable an accurate extraction of keywords from sentences without manipulating statistical scores, barring any typographical errors. Drawing on neurologists’ prior identification of significant clinical keywords, we established a sequential keyword extraction process tailored to abnormal reports. This hierarchical framework serves to streamline our algorithm by minimizing the scope of target variables within the reports. We contend that the reduced complexity of our algorithm is conducive to achieving high performance, particularly in the detection of cerebral dysfunction, focal or generalized seizures, and the identification of IEDs.

However, during internal validation, we encountered a limitation: the keyword of *generalized* was not extracted from the two abnormal EEG reports of patients diagnosed with absence seizures. The generalized 3 Hz spike-and-wave complexes represent the typical electrographic signatures of absence seizures (25, 26). Consequently, although the clinical correlations observed in the abnormal reports of patients with absence seizures are not universally applicable, these reports strongly suggest the presence of generalized seizures based on their signatures. To address this challenge, it may be beneficial to implement deep learning techniques aimed at analyzing the reports at the sentence level, or to introduce supplementary rules to accurately identify the type of seizure. To overcome this issue, we may need to utilize specific deep learning-based models to automatically match a variety of regional terminologies for their corresponding channel names.

A significant number of failures were recorded during the final step of Step 2, particularly regarding the localization of IEDs in the abnormal reports from both medical institutions. The present algorithm struggled to detect channel names as the locations of IEDs were frequently described using regional terminologies, such as “*centro-temporal*” or “*fronto-central*,” in 10 abnormal reports (4 from SNUBH and 6 from SNUCH). Thus, these regional terms need to be included as target keywords; however, we are concerned about the vast array of potential combinations of these regional names.

Additionally, our algorithm erroneously identified the channel names that referred to background activities, such as “*delta activities from P4O2*,” in 17 abnormal reports (1 from SNUBH and 16 from SNUCH). Abnormal reports can contain both background activities and IEDs simultaneously. Therefore, it is crucial to develop precise rules for determining channel names after categorizing the data into distinct domains, such as background activity or IEDs. This approach is reminiscent of a previous study by (27), which demonstrated an effective two-level keyword extraction approach from clinical notes. Typographical errors and inappropriate words constituted critical issues that require resolution in our keyword extraction procedures, such as misspelled *generalized* and *delta waves* instead of *delta activities* in the abnormal reports from SNUBH and SNUCH. In the rule-based keyword extraction, it may be highly challenging to overcome those troubles due to the necessity of additional complicated rules for searching replaceable words based on the detection of every possible types of typos or approximate string matching.

During the internal and external validation procedures, typographical errors and improper word usage could have led to both false negatives and false positives. For example, EEG reports that misspelled the term *generalized* have been mistakenly classified as lacking generalized seizure characteristics. Similarly, reports noting *delta waves* as background activity could have been erroneously interpreted as containing IEDs simply due to the appearance of the word *waves*. However, because these observations were drawn from only a few instances, it remains difficult to make broad generalizations based solely on these examples. Moving forward, we plan to build large-scale databases comprising numerous EEG reports to more thoroughly investigate and address such errors, ultimately improving the reliability and accuracy of automated EEG report analysis.

### 4.3 Applications and limitations

A multitude of studies leveraging NLP techniques have focused on the analysis of unstructured data related to epilepsy. Rule-based methodologies have been effectively employed to extract various seizure-related parameters (8–11, 13), as well as patient clinical information (8) and distinct epilepsy phenotypes (7) from clinical notes and discharge summaries. Additionally, BERT-based models have been implemented to categorize clinical notes based on seizure occurrence, achieving a notable median accuracy of 83.7% (11). These models have also been applied to classify publicly available clinical reports with respect to epilepsy and related abnormalities, resulting in impressive F1 scores of 82 and 97%, respectively (12).

In a recent advancement, a transformer-based large language model known as clinical-longformer, pre-trained on publicly accessible clinical notes, was utilized to predict seizure recurrence in EMR data, achieving an F1 score of 82.6% (28). Note that, to date, no NLP-focused studies have successfully developed a method for transforming unstructured EEG reports into structured clinical components. We posit that our hierarchical algorithm could serve a critical function in the establishment of specialized databases, facilitating the organization and analysis of a significant number of EEG reports from patients diagnosed with epilepsy. Unlike the visual interface based on discharge summaries reported in a previous study (13), our structured outcomes in a tabular format required specific keywords solely from EEG reports. Table 4 presents a comparative overview of the performance metrics for our method alongside those reported in the aforementioned NLP studies. While these comparisons can provide useful insights, making direct parallels is challenging due to the distinct objectives and methodologies employed in each investigation. Importantly, the novelty of our work lies in the development of structured datasets derived from EEG reports—an underutilized resource in clinical research—that extend beyond conventional NLP techniques. By focusing on this unique data source, our approach has the potential to enhance clinical databases in ways that previous studies have not fully explored, thereby paving the way for more comprehensive and clinically relevant analyses. By integrating NLP techniques with large-scale medical records—such as discharge summaries and clinical notes—researchers and clinicians can enhance diagnostic processes for complex conditions, including rare and previously undiagnosed diseases. These approaches enable the automated extraction and analysis of relevant clinical information, potentially improving both the speed and accuracy of identifying elusive disorders that often pose significant challenges to traditional diagnostic methods (29). It could also potentially aid with tailored treatment and diagnosis of clinical diseases with text-heavy clinical notes such as headache patients (30).

**Table 4:** Comparison of performance metrics of our work and recent natural language processing studies in the field of epilepsy.

Study	Method	Objective	Result
This study	Rule-based and BERT	To convert EEG reports into tabular data by classification and keyword extraction	• Internal: accuracy = 0.985 (focal or generalized seizure), accuracy = 0.975 (identification of IEDs and locations) • External: accuracy = 1.0 (focal or generalized seizure), accuracy = 0.885 (identification of IEDs and locations)
Beaulieu-Jones et al. (28)	Clinical-longformer	To predict seizure recurrence after an initial seizure-like event	• Additional domain-specific and location-specific pretraining: F1-score = 0.826, AUC = 0.897 • No pretraining: F1-score = 0.739, AUC = 0.846
Tao et al. (9)	Rule-based	To extract temporal information of seizure onset from discharge summaries	Precision = 0.750, recall = 0.651, and F1-score = 0.697
Xie et al. (11)	BERT	To extract clinical information (seizure frequency, seizure freedom) from clinical notes	• Median accuracy for classification: 0.837 (BioClinical BERT), 0.747 (RoBERTa) • Median F1 score for text extraction: 0.845 and 0.834 (RoBERTa)
Decker et al. (10)	Rule-based	To extract seizure data (seizures and frequency) from clinical notes	• Internal test: recall = 0.70, precision = 0.95, and F1-score = 0.82 • External test: recall = 0.22, precision = 0.73, and F1-score = 0.40
Rawal and Varatharajah (12)	Rule-based and BERT	To extract attributes for organized reporting from EEG reports	• Seizure classification: F1-scores = 0.92 • Epilepsy classification: F1-scores = 0.82 • Normal and abnormal classification: F1-scores = 0.97
Fonferko-Shadrach et al. (8)	Rule-based	To extract detailed clinical information from epilepsy clinic letters	Precision = 0.914, recall = 0.814, and F1-score = 0.861
Cui et al. (7)	Rule-based	To extract epilepsy phenotypes and anatomical locations from clinical discharge summaries	• Epilepsy phenotypes: micro-averaged precision = 0.924, recall = 0.931, and F1-score = 0.927 • Correlated phenotypes and anatomical locations: precision = 0.852, recall = 0.859, and F1-score = 0.856
Cui et al. (13)	Rule-based	To extract seizure-related clinical free text from discharge summaries	Precision = 0.936, recall = 0.840, and F1-score = 0.885

Thus, our methodology presents an innovative NLP-driven framework aimed at extracting pertinent information from unstructured text within medical reports, specifically targeting EEG reports. We customized our algorithm to align with the unique formatting of these reports, facilitating the extraction of key variables of interest. Nevertheless, this study acknowledges several notable limitations. First, the internal and external validation phases were conducted using a relatively small sample of EEG reports, which may impact the robustness of our findings. Additionally, the EEG reports utilized in the external validation phase bore a close resemblance to those in the internal validation, thereby limiting the diversity of our dataset. To enhance the validity and generalizability of our findings, we strongly advocate for extensive multi-institutional studies that can address these concerns regarding sample size and diversity. Our study was constrained by the use of internal and external datasets that were relatively similar, which may have limited the generalizability of our findings. To address this issue and improve the robustness of our text classification models, we could incorporate EEG reports from multiple institutions representing various reporting formats and clinical settings. By doing so, we anticipate not only enhancing the performance of our classifiers but also developing more targeted keyword extraction strategies tailored to each institution’s unique report structure, ultimately leading to more accurate and widely applicable analysis of EEG data in the future studies. In terms of ethical concerns and data security, it is highly required to ensure that all the reports have no patients’ personal and sensitive medical records keeping them safe in their corresponding institutions. In terms of data access, federated learning can be suggested for deep learning and NLP tasks through multi-institutional collaborations (31). Also, future research should consider exploring state-of-the-art large language models beyond the present rule-based methods for tackling complex tasks such as handling typographical errors, inappropriate words, and regional terminologies by automated correction techniques (32, 33).

## 5 Conclusion

This study introduces a hierarchical algorithm designed to transform unstructured EEG reports from pediatric epilepsy patients into structured data presented in a tabular format through the application of NLP techniques. Utilizing BERT-based deep learning models for text classification, we subsequently applied a series of rule-based procedures for the extraction of relevant keywords. Given that neurologists pre-select specific clinical keywords, we crafted a hierarchical structure that streamlines the process, enhancing the ability of the algorithm to manage the nuances of free-text EEG reports and produce standardized tables. We believe that our approach holds significant promise for the creation of specialized databases focused on EEG reports, thereby advancing healthcare research and clinical applications.

## Data Availability

The original contributions presented in the study are included in the article/Supplementary material, further inquiries can be directed to the corresponding author.
