# Supplementary material for: Data transformation of unstructured electroencephalography reports by natural language processing: improving data usability for large-scale epilepsy studies
Source: Front Neurol. 2025 Feb 28;16:1521001. doi: 10.3389/fneur.2025.1521001 (PMC11906308; doi:10.3389/fneur.2025.1521001)
Supplement: Supplementary file 1 [file Supplementary_file_1.docx]

Supplementary Material

# Supplementary Figures and Tables

## Supplementary Figures

**Supplementary Figure 1.** Model architectures of BERT with LSTM and Clinical BERT with LSTM. Clinical BERT is a specialized variant of BERT pretrained on clinical text corpora which has a structure same as BERT.

| 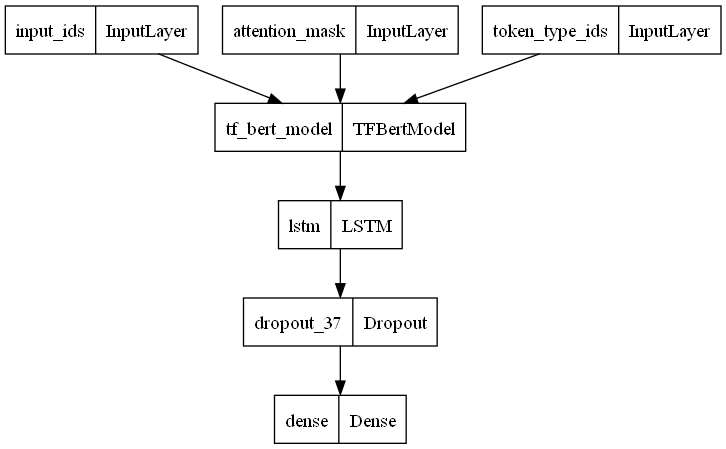 | | | |
| --- | --- | --- | --- |
| Layer (type) | Output Shape | Param # | Connected to |
| input_ids (InputLayer) | [(None, 128)] | 0 | [ ] |
| attention_mask (InputLayer) | [(None, 128)] | 0 | [ ] |
| token_type_ids (InputLayer) | [(None, 128)] | 0 | [ ] |
| tf_bert_model (TFBertModel) | TFBaseModelOutput With Pooling And Cross Attentions  (last_hidden_state=(None, 128, 768), pooler_output=(None, 768), past_key_values=None, hidden_states=None, attentions=None, cross_attentions=None) | 108310272 | ['input_ids[0][0]', 'attention_mask[0][0]', 'token_type_ids[0][0]'] |
| lstm (LSTM) | (None, 64) | 213248 | ['tf_bert_model[0][0]'] |
| dropout_37 (Dropout) | (None, 64) | 0 | ['lstm[0][0]'] |
| dense (Dense) | (None, 1) | 65 | ['dropout_37[0][0]'] |
| Total params: 108,523,585  Trainable params: 108,523,585  Non-trainable params: 0 | | | |

## Supplementary Tables

**Supplementary Table 1.** An EEG report is tokenized into 69 tokens using AutoTokenizer from Hugging Face. Then, it is converted to an input array with a length of 128 using zero padding and truncation.

| EEG report | Impression) this is a moderately abnormal sleep record due to nearly continuous high amplitude 4-5 Hz theta bursts intermixed with repetitive spike discharges from both frontal area, followed by brief attenuation of whole background activity  Clinical correlation: this record is indicative of diffuse cerebral dysfunction and consistent with partial seizure |
| --- | --- |
| Tokenized  (Token length = 69) | input ids : [101, 8351, 114, 1142, 1110, 170, 19455, 22832, 2946, 1647, 1496, 1106, 2212, 6803, 1344, 25437, 125, 118, 126, 177, 1584, 1103, 1777, 21254, 9455, 3080, 14771, 1114, 26976, 21644, 12398, 1116, 1121, 1241, 22172, 1298, 117, 1723, 1118, 4094, 1120, 5208, 10255, 1104, 2006, 3582, 3246, 7300, 18741, 131, 1142, 1647, 1110, 1107, 26289, 1104, 4267, 3101, 5613, 21831, 173, 6834, 26420, 1105, 8080, 1114, 7597, 20752, 102] |
|  | ['[CLS]', 'impression', ')', 'this', 'is', 'a', 'moderately', 'abnormal', 'sleep', 'record', 'due', 'to', 'nearly', 'continuous', 'high', 'amplitude', '4', '-', '5', 'h', '##z', 'the', '##ta', 'bursts', 'inter', '##mi', '##xed', 'with', 'repetitive', 'spike', 'discharge', '##s', 'from', 'both', 'frontal', 'area', ',', 'followed', 'by', 'brief', 'at', '##ten', '##uation', 'of', 'whole', 'background', 'activity', 'clinical', 'correlation', ':', 'this', 'record', 'is', 'in', '##dicative', 'of', 'di', '##ff', '##use', 'cerebral', 'd', '##ys', '##function', 'and', 'consistent', 'with', 'partial', 'seizure', '[SEP]'] |
| Input array  (Input shape = (1, 128)) | array([[101, 8351, 114, 1142, 1110, 170, 19455, 22832, 2946, 1647, 1496, 1106, 2212, 6803, 1344, 25437, 125, 118, 126, 177, 1584, 1103, 1777, 21254, 9455, 3080, 14771, 1114, 26976, 21644, 12398, 1116, 1121, 1241, 22172, 1298, 117, 1723, 1118, 4094, 1120, 5208, 10255, 1104, 2006, 3582, 3246, 7300, 18741, 131, 1142, 1647, 1110, 1107, 26289, 1104, 4267, 3101, 5613, 21831, 173, 6834, 26420, 1105, 8080, 1114, 7597, 20752, 102, 0, 0, 0, 0, 0, 0, 0, 0, 0, 0, 0, 0, 0, 0, 0, 0, 0, 0, 0, 0, 0, 0, 0, 0, 0, 0, 0, 0, 0, 0, 0, 0, 0, 0, 0, 0, 0, 0, 0, 0, 0, 0, 0, 0, 0, 0, 0, 0, 0, 0, 0, 0, 0, 0, 0, 0, 0, 0, 0]]) |

**Supplementary Table 2.** Detailed outputs of the Clinical BERT with LSTM model alongside the corresponding keyword extraction results for 1,000 normal and 1,000 abnormal EEG reports (excel file).

**Supplementary Table 3.** Confusion matrices over 6-fold cross-validation of the Clinical BERT with LSTM model. Among 6,173 normal and 6,173 abnormal EEG reports, 1,000 normal and 1,000 abnormal reports were used for evaluation and the remaining 5,173 normal and 5,173 abnormal reports were used for model training in each round. TN, TP, FP, and FN denote true negative, true positive, false positive, and false negative, respectively.

| Round |  |  | Predicted | |  | Number of EEG reports | | | |
| --- | --- | --- | --- | --- | --- | --- | --- | --- | --- |
|  |  |  | Normal | Abnormal |  | TN | TP | FP | FN |
| 1 | True | Normal | 1,000 | 0 |  | 1,000 | 999 | 0 | 1 |
|  |  | Abnormal | 1 | 999 |  |  |  |  |  |
| 2 | True | Normal | 1,000 | 0 |  | 1,000 | 998 | 0 | 2 |
|  |  | Abnormal | 2 | 998 |  |  |  |  |  |
| 3 | True | Normal | 1,000 | 0 |  | 1,000 | 999 | 0 | 1 |
|  |  | Abnormal | 1 | 999 |  |  |  |  |  |
| 4 | True | Normal | 1,000 | 0 |  | 1,000 | 999 | 0 | 1 |
|  |  | Abnormal | 1 | 999 |  |  |  |  |  |
| 5 | True | Normal | 1,000 | 0 |  | 1,000 | 999 | 0 | 1 |
|  |  | Abnormal | 1 | 999 |  |  |  |  |  |
| 6 | True | Normal | 999 | 1 |  | 999 | 999 | 1 | 1 |
|  |  | Abnormal | 1 | 999 |  |  |  |  |  |
